# Screening programs for common maternal mental health disorders among perinatal women: report of the systematic review of evidence

**DOI:** 10.1186/s12888-022-03694-9

**Published:** 2022-01-24

**Authors:** Ahmed Waqas, Ahmreen Koukab, Hafsa Meraj, Tarun Dua, Neerja Chowdhary, Batool Fatima, Atif Rahman

**Affiliations:** 1 Institute of Population Health, University of Liverpool, Liverpool, L69 3GF UK; 2 Human Development Research Foundation, Islamabad, Pakistan; 3 Department of Mental Health and Substance Use, World Health Organization, Geneva, Switzerland

## Abstract

Postpartum depression and anxiety are highly prevalent worldwide. Fisher et al., estimated the prevalence of depression and anxiety at 15.6% during the antenatal and 19.8% during the postpartum period. Their impact on maternal and child health is well-recognized among the public health community, accounting for high societal costs. The public health impact of these conditions has highlighted the need to focus on the development and provision of effective prevention and treatment strategies.

In recent decades, some advances have been made in the development of effective universal and targeted screening programmes for perinatal depression and anxiety disorders. Recent research has shown potential benefits of universal and targeted screening for perinatal depression, to identify and treat undiagnosed cases, and help thwart its deleterious consequences. Ethical implications, however, for these screening programmes, without the provision of treatment have often been emphasized.

The present mixed-methods systematic review and meta-analysis was conducted to collate evidence for screening programmes for perinatal depression and anxiety. It aims to answer the following questions, in a global context: For women in the perinatal period, do screening programmes for perinatal depression and anxiety compared with no screening improve maternal mental health and infant outcomes?

A series of meta-analyses reveal a reduction in perinatal depression and anxiety among perinatal women undergoing screening programmes. For the outcome of depressive disorder, meta-analysis indicates a positive impact in favour of the intervention group (OR = 0.55, 95% CI: 0.45 to 0.66, *n* = 9009), with moderate quality of evidence. A significant improvement (high quality) was also observed in symptoms of anxiety among perinatal women (SMD = − 0.18, 95% CI: − 0.25 to − 0.12, *n* = 3654).

**Supplementary Information:**

The online version contains supplementary material available at 10.1186/s12888-022-03694-9.

## Introduction

Pregnancy is generally viewed as a rewarding experience and marks the beginning of a woman’s new social role as a mother. However, for some women, it can be a stressful event [1], associated with cultural stigmas, socioeconomic stressors, and gender discrimination in some societies. Pregnant women and expectant mothers undergoing distress often experience varying degrees of emotional dysregulation [2]. It may also trigger the onset of common mental disorders (CMDs) especially depression and anxiety during the perinatal period [2].

Anxiety and depression during the perinatal period are leading causes of disability in women around the world [3]. Their prevalence is estimated at 13% in high-income (HIC) and 19.8% in low-and-middle income countries (LMIC) [4]. In the United States alone, 1 in 7 pregnant and perinatal women suffer from anxiety and depressive disorders. Despite its high prevalence in the US, only half of these women seek mental healthcare due to fear of stigma, or poor mental health awareness. According to Luca et al., the societal costs of these CMDs in the US alone were over 14 billion USD for all births in 2017 [5].

Perinatal anxiety and depressive disorders are major global health concerns, due to their deleterious maternal and child consequences. Mothers with perinatal depression and anxiety often experience social withdrawal from their social networks and foster a poorer relationship with their neonates [6, 7]. They may also experience thoughts of poor self-esteem, self-harm, and suicidal ideation and thoughts about harming the child [8]. Children born to mothers with perinatal anxiety and depression often report poor cognitive, motor and language development, behavioral disorders, and poor academic performance [9, 10]. These conditions are also associated with high infant infection rates and hospitalizations and have a higher risk of preterm birth, low birth weight and poor physical growth [3, 11]. Thus, perinatal common mental disorders place the developing children at a disadvantage right from their birth, contributing to a vicious cycle of disparity extending across generations [12].

The global health impact of perinatal depression and anxiety is well-recognized among the public health community and there is a growing interest in devising prevention and treatment strategies for them, both in HICs and LMICs. Recent research has shown potential benefits of universal and targeted screening for perinatal depression, to identify undiagnosed cases and subsequently treat it [13], and help thwart its deleterious consequences. Ethical implications of national screening programmes (universal or targeted) without provision of treatment have often been emphasized [14] by national and international organizations around the world.

Screening for perinatal depression and anxiety is usually performed using valid and reliable psychometric scales. Most frequently used psychometric scales include the Edinburgh Postnatal Depression Scale (EPDS) and the Patient Health Questionnaire-9 items (Table 1). These scales have been tested and found valid for screening perinatal depression around the globe and in a variety of clinical and community settings. Besides these scales, short screeners comprising of two items such as the Whooley questions are also available for use in busier settings [8]. A summary of these tools is provided in Table 1. Despite their usefulness, these scales also have some shortcomings including heterogeneity across different scales, conflict with DSM criteria of diagnoses, and distress due to misclassifications [15, 16].

**Table 1:** Psychometric scales for screening of perinatal depression

Scale	Detail	Items	Response	Symptoms assessed
Edinburgh Postnatal Depression Scale	A tool that assists health professionals in screen for depressive symptoms among postpartum women. It was developed in the UK by Cox et al., in 1987^a^.	10 items	Likert scale ranging from 0 (not at all) to 3 (all the time)	∙ Mood reactivity ∙ Anhedonia ∙ Self-blame ∙ Anxious ∙ Feelings of panic ∙ Coping ability ∙ Difficulty in sleeping ∙ Feelings of sadness ∙ Crying episodes ∙ Self-harm
Patient Health Questionnaire-9 items	A tool for assessing major depressive disorder in primary care settings. It is based on the DSM-IV for diagnosis of major depressive disorder^b^.	9 items	Likert scale ranging from 0 (not at all) to 3 (nearly every day)	∙ Anhedonia ∙ Low mood and hopelessness ∙ Insomnia or hypersomnia ∙ Fatigue ∙ Poor appetite or hyperphagia ∙ Poor self-esteem ∙ Lack of concentration ∙ Psychomotor retardation or agitation ∙ Suicidal ideation/self-harm
Whooley questions	Two item screeners for perinatal depression recommended by the National Institute of Healthcare Excellence in the UK, for use in busier settings^c^.	2 items	Dichotomous (Yes/No)	∙ Depressed/hopeless ∙ Anhedonia

^a^Cox JL, Holden JM, Sagovsky R. Detection of postnatal depression: development of the 10-item Edinburgh Postnatal Depression Scale. The British journal of psychiatry. 1987 Jun;150(6):782–6

^b^Spitzer RL, Kroenke K, Williams JBW, and the Patient Health Questionnaire Study Group. Validity and utility of a self-report version of PRIME-MD: the PHQ Primary Care Study. JAMA.1999;282:1737–1744

^c^Whooley MA, Avins AL, Miranda J, Browner WS. Casefinding instruments for depression: two questions are as good as many. J Gen Intern Med. 1997;12:439–445

A plethora of primary and secondary research has been published on the psychometric properties of scales for perinatal depression. However, there is a lack of primary research and evidence synthesis efforts on largescale screening programmes especially for LMIC [17–19]. Furthermore, guideline processes in the high-income countries have often resulted in different recommendations for perinatal screening, primarily due to a national focus of guidelines and different methodologies adopted [18, 20]. According to the United States Preventive Services Taskforce, screening programs should only be conducted when there are significant resources for screening, effective treatment, and follow-up [19]. Similar recommendations were given by The National Institute for Health and Care Excellence in the UK [21]. In contrast, the Canadian Task Force on Preventive Healthcare recommended against screening for perinatal depression, due to paucity of evidence [22]. The American College of Obstetrics and Gynaecology recommended that depression screening programs should be “strongly considered” despite the lack of evidence while the American Academy of Pediatrics recommended it at child well-visits, 1, 2, 4 and 6 months perinatal [21, 23].

The aforementioned evidence synthesis efforts have been conducted in the context of HICs and there is a paucity of data for LMICs, therefore, this review aims to answer the following questions, in a global context:

Research question: For women in the perinatal period, do screening programmes (coupled with treatment resources) for common mental health disorders i.e. depression and anxiety compared with no screening improve perinatal maternal mental health and infant outcomes, both in healthcare and community settings?

## Methods

### Database Searches

This systematic review has been conducted according to PRISMA guidelines [24]. Before conduct of this review, its protocol was registered in the PROSPERO database (CRD42020166541) [25]. Using a predefined search strategy (Table 2), an electronic search was conducted in PubMed, Web of Science (including MEDLINE), CINAHL, PsycINFO, Cochrane Central Register of Controlled Trials (CENTRAL), and Global Health Library, in December 2019. We also manually searched bibliographies of eligible full texts and previous guidelines on screening for postpartum depression including the U.S. Preventive Services Task Force recommendation statement [19] and the Agency for Healthcare Research and Quality guidelines [20]. No restrictions were applied for the year of publication or language of studies.

**Table 2:** Search strategy adapted for *pubmed *database

Concept	Keywords
**Condition/population**	(“perinatal depression”[ti/ab] OR “postnatal depression”[ti/ab] OR “postpartum depression”[ti/ab] OR “postnatal anxiety”[ti/ab] OR “postpartum anxiety”[ti/ab] OR “perinatal anxiety”[ti/ab] OR “Depression, postpartum”[MeSh] OR “new mother*”)
**Type of study**	(effectiveness [ti/ab] OR trial*[ti/ab] OR “clinical trial”[ti/ab] OR RCT [ti/ab] OR “randomized clinical”[ti/ab] OR implementation OR evaluation [ti/ab] OR “implementation science” OR feasibility [ti/ab] OR “program development”[ti/ab] OR Fidelity [ti/ab] OR appropriateness [ti/ab] OR acceptability [ti/ab] OR adoption [ti/ab] OR sustainability [ti/ab] OR penetration [ti/ab] OR appropriateness [ti/ab] OR cost-effectiveness)
**Interventions**	(Screen*[ti/ab] OR diagnos* OR detect* OR predict* OR aware* OR identif* OR “mass screening”[MeSh] OR diagnosis[MeSh] OR “Psychodiagnosis” OR “Psychodiagnostic Interview” OR scale OR questionnaire* OR checklist*)
**Maternal Outcomes**	(sensitivity OR specificity OR “maternal mortality” OR anaemia OR anemia OR “back pain” OR “breast complications” OR fatigue OR tiredness OR exhaustion OR “sleep deprivation” OR “weight retention” OR well-being OR self-esteem OR stress OR anxiety OR depression OR self-harm OR suicide OR “intimate partner violence” OR “readmission to hospital” OR “length of stay” OR “need of medication” OR “Maternal functioning” OR “emotional attachment” OR self-efficacy OR competence OR autonomy OR confidence OR self-care OR “coping skills” OR “infant care” OR “mother-child interactions” OR “daily living” OR “social support” OR “quality of life” OR “responsive care giving” OR “neonatal mortality” OR infection OR sepsis OR omphalitis OR jaundice OR disability OR allergy OR surgery OR injury OR immunization OR growth OR height OR weight OR “head circumference” OR “motor development” OR “developmental milestone*” OR breastfeeding)

### Inclusion & Exclusion criteria

For this review, the effectiveness of screening programs for perinatal anxiety and depression was assessed using data from randomized controlled trials, cluster randomized controlled trials, and cross-over trials. Implementation processes, acceptability, and feasibility of screening programmes were assessed using qualitative and mixed-method studies. To ensure the inclusion of the latest evidence and more recent definitions of and diagnostic criteria for postpartum depression and anxiety, studies published in the last twenty years (the year 2000 to 2019) were considered.

Only those trials were considered that screened women during the antenatal period till 12 months postpartum. We excluded those studies which focused on postpartum blues described as transient or mild depressive symptoms after delivery, not meeting the DSM or ICD diagnostic criteria for perinatal depression.

Only those screening programs were included that comprised of a minimum set of sequential processes and elements especially the presence of treatment and referral options post-screening, as outlined in Public Health England’s recommendations [26]. In terms of screening tests specific for perinatal depression and anxiety, these elements included: a screening test for case identification using a psychometrically validated scale with a defined cut-off score (such as EPDS and PHQ-9); information about test results followed by a diagnostic interview to ascertain diagnoses; management options by taking into consideration women’s psychosocial context and provision of treatment resources for women opting for treatment. Studies reporting only rates of depression management or treatment post-screening and not outcomes associated with depression were not considered. We also considered qualitative or mixed methods studies reporting acceptability, feasibility, and attitudes toward and cost-effectiveness of screening programmes.

### Primary outcomes

Quantitative evidence for the effectiveness of screening instruments was assessed across four primary outcomes selected apriori: severity of perinatal (a) anxiety or (b) depression measured using validated psychometric instruments b) rates of perinatal (c) anxiety or (d) depression assessed using the International Classification of Diseases or Diagnostic Statistical Manual criteria of diagnoses.

### Secondary outcomes

Several secondary outcomes were selected apriori, pertaining to maternal physical morbidity and psychosocial functioning as well as infant physical and cognitive health. These secondary outcomes included:

### Maternal outcomes

- Psychosocial distress
- Pattern of health services utilization
- Quality of life
- Maternal physical morbidities
- Quality of maternal-infant attachment or bonding
- Rates of exclusive and continuous breastfeeding.

### Infant outcomes

- Cognitive health
- Physical health

### Implementation processes, acceptability, and feasibility

- Cost-effectiveness
- Barriers and facilitators to uptake of these interventions assessed using qualitative interviews or mixed-method study designs.

### Screening of bibliographic records & data extraction

Selection of eligible studies was done by two independent investigators (HM & AK), firstly by a screening of titles and abstracts and then by scrutinizing full texts of studies. This process was mediated by a senior investigator (AW) in case of any differences or discrepancies between the reviewers. Data extraction was performed across several matrices including characteristics of population and screening programmes especially timing, setting, type of psychometric tools and their cut-off values, and type of treatment offered to women who screened positive for perinatal depression. Acceptability and uptake of screening programs were assessed by extracting data across relevant outcomes reported in studies with qualitative or mixed-method study designs. This was done by extracting quantitative data or quotes of participants or interpretation of researchers.

### Risk of bias (quality) assessment

The risk of bias in the conduct of RCTs was assessed using the Cochrane tool for risk of bias assessments, across randomization procedures, method for allocation concealment, blinding of participants and personnel and outcome assessors, attrition bias, and other biases [27]. Since it is relatively difficult to blind participants and personnel in psychological interventions, we coded this dimension as having a high risk for all studies.

### Strategy for data synthesis

For meta-analysis across quantitative outcomes, pooled effect sizes were calculated using post-intervention mean, standard deviation, and sample sizes for quantitative outcomes. For binary outcomes, we used post-intervention number of events and sample sizes for both the intervention and control groups. Random effects model was utilized throughout the study due to expected clinical, methodological, and statistical heterogeneity across the studies [28]. Sensitivity analyses were conducted to assess the contribution of each study toward pooled effect size. Publication bias was assessed for outcomes reported in more than five studies, using Begg’s funnel plots and Egger’s regression [29]. Subgroup analyses were conducted when specific subgroups were reported in more than four studies and meta-regression analyses for continuous moderators reported in ten studies [30, 31]. Qualitative studies and mixed-method studies were synthesized for assessing the implementation process, using a narrative synthesis strategy [25].

### GRADE profile

GRADE evidence criteria were used to gauge the quality of evidence from very low to high based on several criteria including the risk of bias, indirectness, imprecision, inconsistency, publication bias, and dose-response relationship [32].

### Description of narrative review methodology

Along with the appraisal of quantitative evidence, this report also sought to provide a narrative review of qualitative and feasibility studies. The narrative review was conducted to establish acceptability, feasibility, and attitudes toward and cost-effectiveness of screening programmes. For this purpose, two independent coders (AK & HM) reviewed the eligible full texts to extract quantitative or qualitative data relevant to previously mentioned outcomes. After extraction of relevant content, it was categorized based on its scientific content and broader themes in their respective area subsets. Although the broad themes for coding and categorization of this data were defined apriori, we sought to keep the coding approach open during this phase. This analytical approach was utilized to be inclusive of the expansive topic of perinatal mental health and identify subtleties and nuances to draw more robust relationships and inferences.

Three broad themes were used for the classification of qualitative content: a) acceptability and attitude toward screening programmes among intervention recipients, b) acceptability and attitude toward screening programmes among delivery agents and c) attitude toward screening programmes among important stakeholders. Several outcomes such as treatment satisfaction, therapeutic bond with the delivery agent, facilitators, and barriers to uptake were considered.

## Results

### Characteristics of studies

**Fig. 1:**
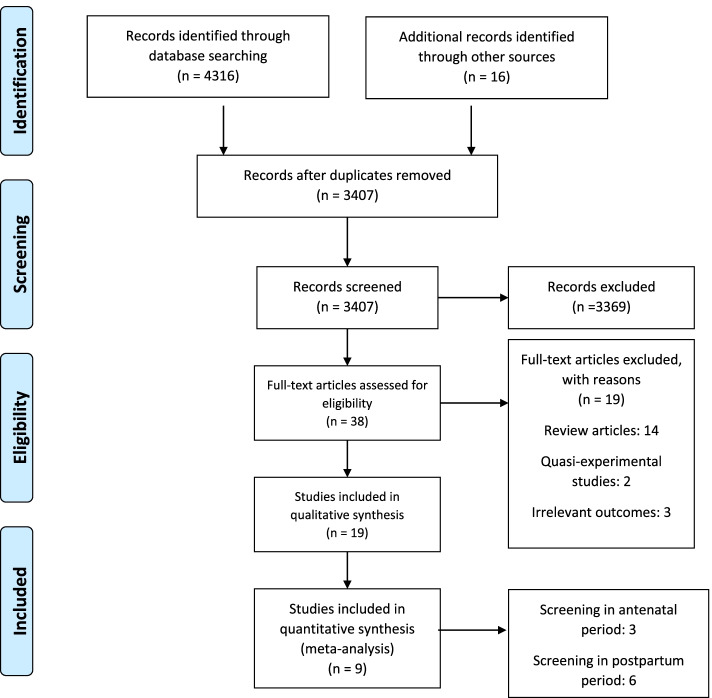
PRISMA flowchart

The database search yielded a total of 4316 studies, which was supplemented by a manual search identifying 16 key studies. A total of 925 duplicate items were removed, with 3407 titles and abstracts screened for eligibility as per our inclusion and exclusion criteria (Fig. 1). Out of 38 full-text articles, we included a total of 19 studies for qualitative synthesis and 9 studies for quantitative synthesis (Fig. 1). Out of these 19 studies, RCT findings were reported in 9 studies (10 trials), cost-effectiveness in 2 studies, and acceptability of screening programmes in 10 studies. Among these, Morrell et al., reported the effectiveness, cost-effectiveness as well as acceptability of a screening programme [13].

### Implementation of screening programs

The quantitative section of this review was informed by three cluster RCTs [13, 33, 34], three RCTs [35–37], two quasi-experimental/controlled clinical trials [38, 39], and one cross-over trial [40]. In contrast to other RCTs, Morrell et al., conducted their cRCT in a pragmatic real-world setting [13]. Half of these trials were published before 2010 and all studies were conducted in high-income countries including the USA (*n* = 2), the UK (*n* = 2), and one each in Hong Kong, Netherlands, Australia, Sweden, and Norway. Settings varied with each study and included primary care centers including GP practices, antenatal care or maternal and child healthcare centers, and child wellness centers, and hospitals and home visits [13]. Delivery agents for screening programmes varied from nurses, nurses specializing in public health, midwives, health visitors, psychology students, and physicians (Table 3). Six of the studies reported screening in the postpartum period and three in the antenatal period.

**Table 3:** Characteristics of screening programmes for perinatal depression

Study	Scope	Mode of screening	Timing of screening	Delivery	Delivery agent	Treatment type	Control group status	Primary outcome-mother	Primary outcome-child	Primary time point
Leung SS, 2011 [37]	Universal	EPDS screening	Postpartum 8 weeks	In Person	Nurse	Non-directive counselling by MCH nurses or management by the community psychiatric team for those with high EPDS scores or Suicidal ideation. This was for both the intervention and control group.	Usual practice where nurses carried out clinical assessment.	EPDS > = 10	Body weight at 6 and 18 months; number of hospitalizations and doctor visits	6 months postpartum
der Zee-van den Berg AI,2017 [39]	Universal	Repeated online screening with EPDS	Postpartum 3 weeks	Self-administered/Online	Nurse	For EPDS > = 13 refer the mother to her family practitioner or mental health care professional; for EPDS 9–12 indicating minor depression, home visits by nurses to check coping capability and if suicidal ideation, referral to crisis center.	Newborns visited WCC at the same, regular basis but received no EPDS screening that guided further advice and referral	EPDS > = 13; presence of depression (major or minor) at 9 months postpartum measured with MINI	ASQ-SE	9 months postpartum
MacArthur C, 2002 [33]	Universal	EPDS screening	Postpartum (28 days)	In Person	Midwives	Care plans were made, and visits scheduled based on these results at least every 28 days so that care could be tailored to individual, GP referrals		Physical and mental component scores of SF36 and EPDS		4 months
Morrell CJ, 2009 [13]	Universal	EPDS screening	6 weeks postpartum	Two groups: In person and postal mail	Health visitors	Cognitive behavioral and person centered (non-directive); SSRI or both SSRI plus CBA/Non-directive for those screened positive on SCAN	Usual care	EPDS score ≥ 12		6 months postpartum
Webster J, 2003 [35]	At risk	EPDS postal	Antenatal	Self-administered	Self	The Educate component of the intervention involved providing women in the intervention group with a booklet about postnatal depression and a list of the phone contacts of postnatal depression resources. The women completed the Edinburgh Postnatal Depression Scale and their risk of developing postnatal depression was discussed with them. In the final part of the intervention (Alert), letters were sent to the women’s referring general practitioner and to their Child Health Nurse with details of their risk status	The control group received standard care, which included case management and referral to a hospital social worker or psychiatrists if required.	rate of depression at four months assessed by the Edinburgh Postnatal Depression Scale.		
Zlotnick, 2006 [36]	At risk	Risk index questionnaire	23–32 weeks’ gestation	In Person	Research team	ROSE Program intervention based on interpersonal therapy: four 60-min group sessions with three to five women assigned to the group over a 4-week period and a 50-min individual booster session after delivery	Routine clinical care	Depression using the BDI		3 months postpartum
Glavin, 2010 [38]	Universal	EPDS	Postpartum six weeks	In Person	Nurses	Active listening and emphatic communication (non-directive counselling): phenomenon and providing information about risk factors, symptoms and the identification of mental health problems and treatment among new mothers; and then referral to mental health team	Care as usual	Depression rates using EPDS		3 months
Wickberg, 2005 [40]	Universal	EPDS	Antenatal	In Person	Self-administered	Non-directive counselling	Care as usual	EPDS scores		36 week antenatal
Yawn, 2012 [34]	Universal	Self-administered EPDS for screening and physician evaluation (using PHQ-9)	Postpartum 5–12 weeks	Postal mail	Two steps: self & Physician	Education and tools for postpartum depression screening, diagnosis, initiation of therapy, and follow-up within their practices	Usual-care practices received a 30-min presentation about postpartum depression	Rates of postpartum depression		12 months

Out of nine studies, six studies reported on training curriculum for these delivery agents for screening of peripartum depression. These included: Lectures on perinatal depression and non-directive counselling; structured reflective practice sessions using role-play, peer supervisory session [13]; general information on postpartum depression, on screening and diagnosis, as well as training and practice of nursing telephone calls, and using PHQ-9 in case studies [34] and different aspects of depression, such as symptoms, aetiology and effects, and about the value of listening and support. Four studies reported psychologists and senior mental health professionals as a supervisory staff [13, 37–39]. Only Morrell et al., reported significant details on supervision, fidelity, and supervision of delivery agents, by employing a training reference group [13].

### Screening strategies

Seven out of ten trials employed the EPDS scale for assessing rates of depression [13, 33, 35–38, 40]. Other scales used for assessment of depression were PHQ-9 [34], Beck Depression Inventory [36], and risk index questionnaire. Several studies also employed Mini-International Neuropsychiatric Interview major depression scale [36], Schedules for Clinical Assessment in Neuropsychiatry interviews, and clinical assessments by physicians and public health nurses as a confirmatory test for peripartum depression [13, 34, 38]. Although a larger proportion of studies conducted an in-person assessment of postpartum depression, other modes included online delivery [39], postal questionnaires [13, 35], and clinical assessment [13, 38]. Varied time points for screening, ranging from 23 to 32 weeks of gestation and four to six weeks after birth, were reported in different studies.

### Treatment Strategies

Non-directive counselling, psychoeducation and pharmacological therapy were the most frequently cited treatment strategies utilized in these trials. Leung et al., offered non-directive counselling by maternal and child health nurses or management by the community psychiatric team for those with high EPDS scores or suicidal ideation [37]. In the study reported by Van der Zee-van et al., the mothers with depression were referred to their family practitioner or mental health care professional; for EPDS score of 9–12 indicating minor depression, home visits were conducted by nurses to check coping capability and if suicidal ideation was present, referral to crisis center were made [39]. In Macarthur et al’s programme, care plans were made and visits scheduled based on these results at least every 28 days so that care could be tailored to individuals supported by GP referrals [33]. Morrel et al., provided cognitive behavioral therapy and person-centered (non-directive) counselling; Selective Serotonin Reuptake Inhibitors (SSRI), or both SSRI plus cognitive behavioural approaches/non-directive for those screened positive on SCAN [13]. Webster et al., sent referral letters to the women’s referring general practitioner and to their child health nurse with details of their risk status [35]. Zlotnick et al., provided group-based interpersonal therapy and individual booster sessions after delivery [36]. Glavin et al., provided with non-directive counselling, psychoeducation and referral to the mental health team [38]. Wikcberg provided non-directive counselling and Yawn et al., provided education and tools for postpartum depression screening, diagnosis, initiation of therapy, and follow-up within their practices [40].

### Meta-analytical Evidence

A series of meta-analyses were conducted to delineate the effectiveness of screening programmes across a range of outcomes (Table 4, Supplementary Figs. 1, 2, 3, 4 and 5).

**Table 4:** Screening programmes compared to care as usual for postpartum depression?

**Patient or population**: Pregnant women and new mothers with symptoms of depression or anxiety **Intervention**: screening programmes **Comparison**: care as usual						
Outcome № of participants (studies)	Relative effect (95% CI)	**Anticipated absolute effects (95% CI)**			Certainty	What happens
				**Difference**		
Rates of depression assessed with: Psychometric scales № of participants: 9009 (10 RCTs)	**OR 0.55** (0.45 to 0.66)	17.5%	**10.4%** (8.7 to 12.3)	**7.0% fewer** (8.8 fewer to 5.2 fewer)	⨁⨁⨁◯ MODERATE ^a^	Screening programmes likely reduces rates of depression slightly.
Severity of Anxiety symptoms assessed with: Psychometric scales № of participants: 3654 (3 RCTs)	–	–	–	SMD **0.18 SD lower** (0.25 lower to 0.12 lower)	⨁⨁⨁⨁ HIGH	Screening programmes reduces severity of Anxiety symptoms slightly.
Treatment seeking № of participants: 1082 (3 RCTs)	**OR 3.74** (2.14 to 6.52)	17.4%	**44.0%** (31 to 57.8)	**26.6% more** (13.7 more to 40.4 more)	⨁⨁⨁◯ MODERATE ^b^	Screening programmes likely results in a large increase in treatment seeking.
Parental distress assessed with: Psychometric scales № of participants: 2336 (5 RCTs)	–	–	–	SMD **0.27 SD lower** (0.39 lower to 0.15 lower)	⨁⨁⨁◯ MODERATE ^c^	Screening programmes likely reduces parental distress slightly.
Quality of life assessed with: Psychometric scales № of participants: 5157 (4 RCTs)	–	–	–	SMD **0.2 SD higher** (0.14 higher to 0.27 higher)	⨁⨁⨁⨁ HIGH	Screening programmes increases quality of life slightly.
*The risk in the intervention group (and its 95% confidence interval) is based on the assumed risk in the comparison group and the **relative effect** of the intervention (and its 95% CI). **CI:** Confidence interval; **OR:** Odds ratio; **SMD:** Standardised mean difference						
**GRADE Working Group grades of evidence** **High quality:** We are very confident that the true effect lies close to that of the estimate of the effect **Moderate quality:** We are moderately confident in the effect estimate: The true effect is likely to be close to the estimate of the effect, but there is a possibility that it is substantially different **Low quality:** Our confidence in the effect estimate is limited: The true effect may be substantially different from the estimate of the effect **Very low quality:** We have very little confidence in the effect estimate: The true effect is likely to be substantially different from the estimate of effect						

*Explanations*

a. Three out ten studies were rated as having a overall low risk of bias. Meta-regression did not reveal any significant association of scores on risk of bias scale with the pooled effect size

b. Two out of three studies had an overall higher risk of bias. Subgroup analysis could not be conducted to ascertain association between risk of bias scores and effect size

c. Egger’s regression statistic revealed significant publication bias

#### Postpartum depression

A total of nine studies (10 trials) assessed rates of depressive disorder among pregnant women or postpartum women undergoing screening for perinatal depression. Seven out ten of studies employed the EPDS scale for assessing rates of depression [13, 33, 35–38, 40]. Other scales used for assessment of depression were the MINI major depression scale [36], PHQ-9 [34], and Beck Depression Inventory [36]. The pooled results indicated a positive impact in favour of the intervention group (OR = 0.55, 95% CI: 0.45 to 0.66, *n* = 9009, p=). There was no evidence of significant heterogeneity in reporting of this outcome (I^2^ = 39.75%, Q = 14.94, *P* = 0.09). Removing quasi-experimental study (Van Der Zee-Van Den Berg et al., 2017) from the overall forest plot, did not yield any change in statistical significance. Severity of depressive symptoms was reported by only one study, using the Beck Depression Inventory [36], indicating a non-significant improvement in favour of the intervention group (SMD = − 0.08, 95% CI: − 0.51 to 0.34, *n* = 86).

#### Postpartum anxiety

Three studies reported the severity of anxiety symptoms among the intervention recipients using the State-Trait Anxiety Scale. There was no evidence of heterogeneity in reporting of this outcome (I^2^ = 0%, Q = 1.65, *p* = 0.44). A significant improvement was seen in the intervention group than their counterparts (SMD = − 0.18, 95% CI: − 0.25 to − 0.12, *n* = 3654). Trait anxiety symptoms also improved in favour of intervention group (SMD = − 0.28, 95% CI: − 0.45 to − 0.12, *n* = 565, I^2^ = 0%). Rates of anxiety were not reported in any of the studies.

#### Quality of life

Quality of life was measured across three studies using the Short Form (SF) questionnaire where a greater improvement in the mental component of the SF was reported (SMD = 0.20, 95% CI: 0.14 to 0.27, *n* = 5157, I^2^ = 37.80%). However, no improvement was seen on the physical component of the SF scale (SMD = − 0.03, 95% CI: − 0.23 to 0.17, n = 5157, I^2^ = 0%). Five trials [13, 34, 37, 38] reported scores on the parenting stress index, showing an improvement in stress levels among the experimental group (SMD = − 0.27, 95% CI: − 0.39 to − 0.15, *n* = 2336). An improvement was seen in overall functioning among the experimental group (SMD = 0.35, 95% CI: 0.14 to 0.55, *n* = 373, I^2^ = 0%). Removing quasi-experimental study (Van Der Zee-Van Den Berg et al., 2017) from the overall forest plot, did not yield any change in statistical significance.

#### Treatment-seeking practices

Treatment-seeking practices were reported in three studies [34, 37, 40] as receiving depression treatment or attending referrals, where a significant improvement was reported among women undergoing screening for depression (OR = 3.74, 95% CI: 2.14 to 6.52, *n* = 1082, I^2^ = 52.51%).

#### Marital satisfaction

Women undergoing screening for perinatal depression were more likely to report higher satisfaction levels than their counterparts (SMD = 0.24, 95% CI: 0.14 to 0.35, *n* = 1503). Generally, women in the intervention group reported a non-significant improvement marital/partner satisfaction [34, 37] than their counterparts (SMD = − 0.32, 95% CI: − 0.88 to 0.23, *n* = 1017, I^2^ = 48.23%).

#### Adverse events

Adverse events occurring during the screening programs were mentioned in two studies [13, 37]. The review authors were not able to pool results for adverse effects reported in two studies (4546 women). One trial (462 women) reported no adverse effects in their intervention [13, 37]. Similarly, in the other trial (4084 women) there were no hospital or psychiatric admissions due to adverse events. Also, contacts with other mental health or social workers were rare in the screening group. None of the other trials reported adverse effects of screening programmes for perinatal depression [13, 37].

#### Secondary infant outcomes

Infant outcomes were reported in only three out of nine studies [13, 37, 39]. A weak improvement in child socio-emotional development was reported in the experimental group (SMD = − 0.10, 95% CI: − 0.16 to − 0.04, *n* = 4050, I^2^ = 0%). No improvement was seen among physical development of the infants (SMD = 0.09, 95% CI: − 0.02 to 0.19, *n* = 1486, I^2^ = 0%). Morrell et al., in their trials reported an improvement in parent-child interaction (SMD = 0.32, 95% CI: 0.13 to 0.52, *n* = 565, I^2^ = 26.52%). The number of doctor visits (SMD = 0.19, 95% C: 0.01 to 0.34, *n* = 462) increased among the experimental group, however, no differences were noted in number of hospitalizations (SMD = 0.06, 95% C: − 0.13 to 0.24, *n* = 462).

#### Cost-effectiveness

The cost-effectiveness of screening programmes was evaluated in two studies [13, 41],. Meta-analysis could not be conducted due to varying study designs and methodology assessment.

Wilkinson et al., (2017) reported cost projections in a hypothetical cohort of 1000 pregnant women with one live birth, over a 2-year time horizon [42]. All costs were reported from a Medicaid perspective. Screening for postpartum depression was done face to face and treatment offered was either SSRI (fluoxetine) or IPT delivered by provisionally licensed mental health providers under supervision of a licensed psychiatrist. Compared to usual care, the intervention cost $296,919 more but resulted in an additional 21.43 QALYs and 29 remissions achieved; accounting for an incremental cost-effectiveness ratio of $13,857/QALY gained and $10,182/remission achieved. Using the commonly accepted U.S. willingness to pay a threshold of $50,000 per QALY gained, screening and treating women for postpartum depression was found to be cost-effective [42].

Morrell et al., (2009) conducted cost-effectiveness analyses for their screening programme, embedded in the PoNDER trial [13]. General screening for postpartum depression was done either face to face or through postal questionnaires, by employing health visitors [13]. Women at risk were then interviewed using the SCAN interview schedule. Costing for healthcare needs, screening and treatment was done for mothers at 6 months and then at 12 months for both the mothers and babies. Two types of treatments were offered for women who screened positive for postpartum depression: CBT and non-directive counselling. Those mothers with severe depression and suicidal ideation were referred for psychiatric treatment [13].

Morrell et al., reported that a greater number of QALYs were gained in the intervention group, albeit this increase was non-significant. The greatest increase was reported in the intervention group opting for cognitive behavioral treatment post-screening [13]. This group when compared with their control counterparts or those receiving non-directive counselling was also found to be cost-effective. When QALYs were considered to range between £20,000 and £30,000, the probability for cost-effectiveness was over 70%, for the group of women undergoing cognitive-behavioral treatment, reflecting lower costs and higher QALYs. In addition, the intervention groups reported fewer contacts with health visitors, general practitioners, and social services. Both the control and intervention group reported no mother and baby unit admissions or emergency attendances [13].

#### Publication bias

There was no publication bias (Supplementary Fig. 6) in reporting of the rates of depression (Egger’s regression *p* = 0.18).

#### Risk of bias assessment

**Fig. 2:**
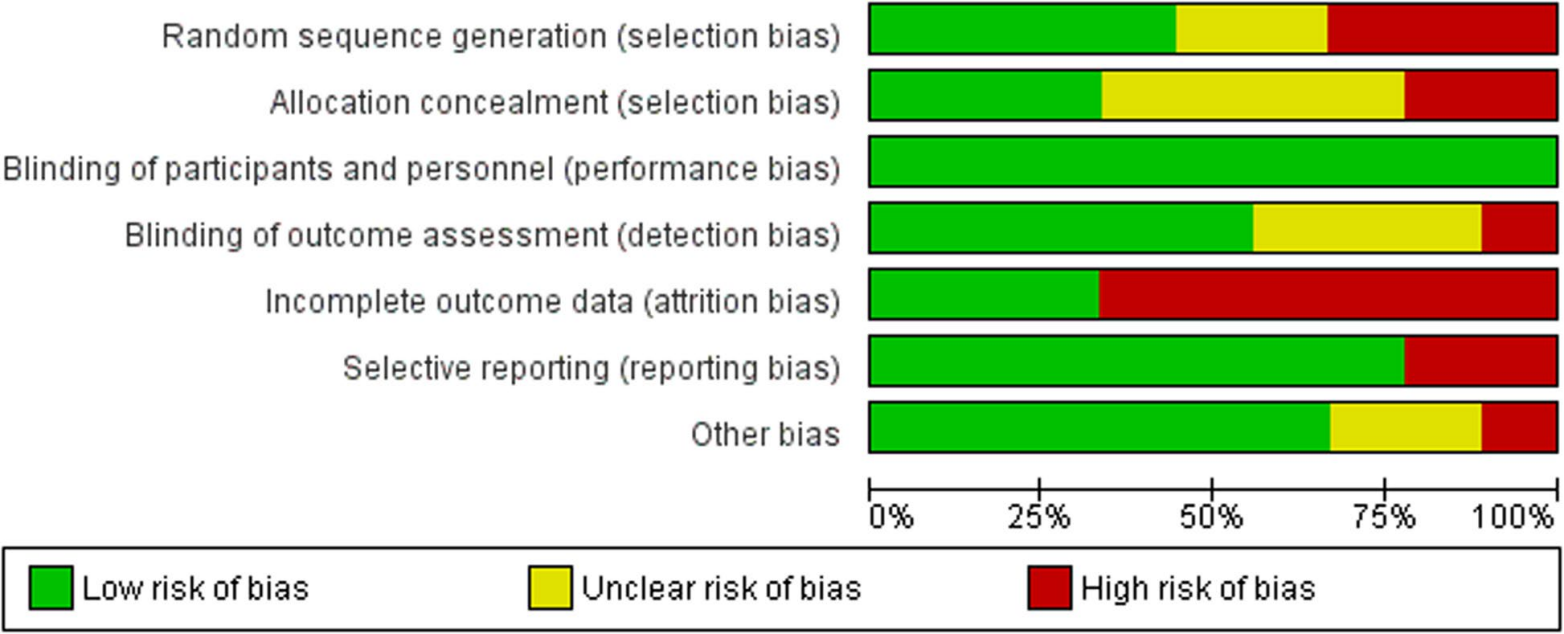
Risk of bias among RCTs assessed with Cochrane tool for risk of bias

Overall, three out of nine studies were of high quality according to the Cochrane Risk of Bias tool [13, 35, 37]. Selection bias and attrition bias were observed in a high proportion of the studies (Fig. 2). Random sequence generation was judged at high/unclear risk of bias in five studies, allocation concealment (*n* = 5), blinding of outcome assessment (*n* = 4), attrition bias (*n* = 6), reporting bias (*n* = 2) and other biases (*n* = 3) (Supplementary Fig. 7).

#### Moderator analysis

Subgroup analysis (Table 5 and Table 6) did not reveal any difference in effect sizes for rates of depression outcome, according to timepoint of screening (postpartum vs antenatal) and type of screening tools and type of treatments offered. Scores on risk of bias scale bore no significant association with effect sizes for rates of depression outcome (*p* = 0.67, R^2^ = 0%).

**Table 5:** Subgroup analysis for the outcome of postpartum depression

Group	No. of studies	Point estimate	95% CI		I^**2**^	Q	***p***
			Lower	Upper			
**Screening timepoint**							
Postpartum	7	−0.36	−0.48	− 0.24	40.71%	0.93	0.34
Antenatal	3	−0.23	− 0.47	0.002	38.29%		
**Tool**							
EPDS	7	−0.31	−0.43	− 0.19	33.09%	4.14	0.25
EPDS & clinical	1	−0.52	−0.81	− 0.24	0		
EPDS & PHQ-9	1	−0.23	−0.49	0.03	0		
Risk index	1	−0.94	−1.85	− 0.03	0		
**Mode of screening**							
In-person interviews	5	−0.39	− 0.57	−0.20	10.55%	0.95	0.81
Postal	1	−0.23	−0.58	0.11	0%		
Multiple methods	2	−0.27	− 0.55	0.01	0%		
Self-administered^a^	2	−0.38	−0.69	− 0.08	88.23%		

^a^Includes online delivery

**Table 6:** Rates of depression according to treatment type offered to women undergoing screening for postpartum depression

Group	No. of studies	Pooled Odds Ratio	I^**2**^	Q	***p***
Cognitive behavioral	1	0.58 (0.36 to 0.94)	0%	4	0.24
Interpersonal	1	0.18 (0.04 to 0.91)	0%		
Non-directive counselling	3	0.60 (0.45 to 0.80)	0%		
Referrals	1	0.81 (0.51 to 1.29)	0%		
Stepped care approach	4	0.47 (0.33 to 0.66)	67.46%		

#### Quality of Evidence

The GRADE approach (Table 4) was used to rate the strength of evidence for primary outcomes of rates of depressive and anxiety disorders and severity of depression and anxiety symptoms. Certainty of evidence for rates of depressive outcomes was rated as moderate, after downgrading it by one level for high risk of bias among eligible RCTs. Although only four out of ten trials were judged as having a lower risk of bias, pooled effect size did not yield any significant association with risk of bias scores. Certainty of evidence for symptoms of anxiety was based on only three, albeit high-quality RCTs. It was judged as having a high-quality of evidence.

In addition, three secondary outcomes were also judged as critical. Treatment-seeking practices and parental distress outcomes were rated as having a moderate quality evidence. The former outcome was downgraded by one level for higher risk of bias among studies, while the latter revealed a significant publication bias. Quality of life outcomes was rated as having a high quality evidence.

#### Acceptability & feasibility of screening programmes: a narrative synthesis

The acceptability of screening programmes was assessed in ten studies [13, 43–49]. These studies employed varying study designs to study the impact and acceptability and feasibility of depression screening programmes. According to study designs, a higher proportion of the studies employed retrospective (*n* = 4), prospective (*n* = 3), and qualitative (*n* = 2) evaluations of screening programmes. These studies were conducted in USA (*n* = 4), Australia (*n* = 3), Singapore (*n* = 1) and UK (*n* = 1). All the studies provided reflections on the acceptability of these programmes by intervention recipients, while providers’ perceptions were reported by only two studies [13, 45]. None of the studies provided perspectives from stakeholders such as policy-makers, technocrats, politicians, and administrators.

*Perceptions of intervention providers* were generally positive for these screening programs. Buist et al., in their evaluation of screening programmes for postpartum depression in Australia, provided providers’ perceptions on the use of EPDS [45]. An overwhelming majority of the screening providers reported that EPDS was easy to use by nurses (83%), midwives (76%) and general practitioners (71%).

*Post-screening programme treatment-seeking practices:* A majority of the studies reported better attitudes and practices toward treatment-seeking practices, among women screened positive for depression. According to Flynn et al., Women undergoing screening more often discussed their depression status with healthcare providers and were more likely to seek treatment for it (39 vs 15%). Seeking care for postpartum depression following screening was explored in two studies [49, 50]. These studies stressed the importance of treatment provision following screening for perinatal depression. Avalos et al., in their prospective evaluation of a screening programme from pre-implementation phase (*n* = 122) to full-implemented stage (*n* = 41,124), reported a corresponding increase in the diagnosis of new cases [47]. In addition, the expected percentage of women receiving treatment increased from 5.9 to 81.9% in this study. Smith et al., however, reported that only a small proportion of their study sample remained in active treatment in primary care, citing the need for further research into the integration of screening programmes in primary healthcare settings [49].

*High compliance & satisfaction:* A positive impact of these screening programs was indicated by a high completion rate than the controls, in most of the studies. For instance, Flynn et al. reported a high compliance rate (95%) among women undergoing routine clinical screening using the EPDS [50]. Patient satisfaction was reported in five studies [44–46, 51, 52]. Satisfaction toward screening programmes ranged from 73.4 to 100%, in these studies.

*Barriers and facilitators:* These satisfaction surveys also sought to identify barriers and facilitators predicting the success of screening programs. Most of the studies explore attitudes towards healthcare professionals either providing screening or treatment for postpartum depression. One of the most frequently explored themes pertained to healthcare providers’ ability to empathize, provision of psychoeducation and help in finding treatment resources [44, 46, 51, 52]. Another important factor for the high acceptability of these programmes was that the screening providers had not labeled, stigmatized, or distressed the mothers [44, 52]. Characteristics of screening providers were explored in greater detail by Morrell et al. Major barriers to woman’s perception of health professionals were openness to emotional issues and ability to validate mother’s feelings rather than concentrating on the baby. In addition, these *listening visits* emphasized a person-centered approach and thus, helped foster a good therapeutic relationship [13]. Morrell & colleagues also hinted that seeking treatment, post-screening from a general practice may be a barrier for some women. This barrier stemmed from the notion that GPs are more suitable for treating physical rather than mental ailments [13].

## Discussion

This critical review collates both the quantitative and qualitative evidence on screening programmes for perinatal depression and anxiety. We could not find any studies reporting screening programmes for perinatal anxiety. We found good quality evidence that women undergoing screening for depression report improved depressive and anxiety symptoms. Only two studies reported mixed findings for long-term infant physical and cognitive health outcomes.

A majority of the screening programmes included in this review employed the EPDS for the assessment of postpartum depression. This was followed by the PHQ-9; both of which are one of the most thoroughly explored tools for the assessment of depressive symptoms among perinatal women. This is also reflected in psychometric investigations, which have also largely focused on the EPDS, and the PHQ-9. This evidence delineating the psychometric properties especially the accuracy of the EPDS and the PHQ-9 have found their utility in heterogeneous populations and settings, albeit, sometimes yielding varied cut-off values across cultures. Previous guidelines by the NICE and the USPSTF also provide detailed evidence for these two scales, for screening of postpartum depression, deeming them suitable for use in primary care and community settings [8, 53].

Comparative analyses (though limited) have found the EPDS and PHQ9 to be comparable. However, a few versions of the PHQ (PHQ-4, PHQ-2, and PHQ-8) yield somewhat lower sensitivity and specificity in the detection of perinatal depression than the EPDS [8, 53]. In busy primary and secondary care settings, however, the NICE recommends that the initial assessment of perinatal depression be conducted using the Whooley questions because of its high sensitivity (~ 100%). Positive responses to the Whooley questions, can then be followed by a detailed assessment using the EPDS or PHQ-9, offering a way to reduce false-negatives.

Present meta-analysis shows that identifying women for the treatment of perinatal depression and anxiety could potentially lead to direct health benefits for women. Timely interventions for perinatal depression could also have indirect health and developmental benefits for their children as noted in several previous studies [3, 54]. This screening could either be done in the community by allied health workers [13] or at the health facility level [34]. This could thus, serve as a means of improving detection, diagnosis and directing women to appropriate treatments [55]. Besides this, to understand the disease burden to plan services and monitor services, it can be implemented at the population level through surveys.

Integrating screening for common mental disorders into primary care and/or maternal and child health services provides many advantages, including more holistic health care, increased accessibility of mental health services for people in need of care, opportunities for reducing the stigma of mental health problems and reduced costs [44, 52]. This recommendation, however, carries several ethical implications. All the studies emphasize that the screening programs should only be conducted when there are resources for screening, diagnosis, effective treatment and follow-up [26]. And practical strategies need to be researched for resource constrained low and middle income countries.

However, these barriers can be overcome by introducing shorter scales such as the 4 item version of the PHQ or community-informant (peers and relatives) based screening strategies [56]. Shorter scales require less intensive training for use especially by the non-specialist health workforce, and less time to administer in busier settings [56]. These can be coupled with task-shifted interventions programmes such as those based on the WHO’s mhGAP guidelines and Thinking Healthy manual. These are evidence-based interventions tailored for maternal mental health that can be delivered by non-specialists [57, 58]. However, it would also be important to provide adequate referral pathways for women who screen positive for severe depression or anxiety and risk of self-harm/suicide or harm to the baby. Moreover, countrywise adaptation of screening tools and training and supervision of health workers to perform screening and subsequent management (whether referral or provision of psychosocial/psychological interventions) is important.

### Strengths & limitations

There are several strengths of this systematic review and meta-analysis. It builds on the strengths of the previous NICE and USPSTF guidelines, and thus, provides holistic evidence on the effectiveness of screening programmes improving perinatal depression and other outcomes of interest. However, the present results should be interpreted with caution. A high proportion of studies were rated as having a high risk of bias in their study designs, thus, threatening their validity. No studies were conducted in the context of low-and lower-middle-income settings, limiting generalizability. In addition to randomized controlled trials, implementation research is required especially in low-and middle-income settings to answer questions related to who does the screening, where, when, and with which tools. No studies were designed specifically for screening perinatal anxiety. There is also a need for research in other mental health problems associated with child-bearing such as psychosis and alcohol/drug use. Only two studies reported infant outcomes and none of the studies conducted long-term followup among children. Additional research into cost-effective screening programmes is needed, especially in the LMIC.

## Supplementary Information

**Additional file 1: Supplementary Figure 1.** Effectiveness of screening programmes in perinatal depression. **Supplementary Figure 2.** Effectiveness of screening programmes in perinatal state anxiety. **Supplementary Figure 3.** Effectiveness of screening programmes in perinatal psychological quality of life. **Supplementary Figure 4.** Effectiveness of screening programmes in perinatal physical quality of life. **Supplementary Figure 5.** Effectiveness of screening programmes in improving treatment seeking behaviours. **Supplementary Figure 6.** Funnel plot visualizing publication bias in reporting of depression outcome. **Supplementary Figure7.** Summary of risk of bias across studies.

## Data Availability

all data associated with this manuscript have been provided in the main text.
