# Supplementary material for: Screening programs for common maternal mental health disorders among perinatal women: report of the systematic review of evidence
Source: BMC Psychiatry. 2022 Jan 24;22:54. doi: 10.1186/s12888-022-03694-9 (PMC8787899; doi:10.1186/s12888-022-03694-9)

Supplementary figure 1: Effectiveness of screening programmes in perinatal depression


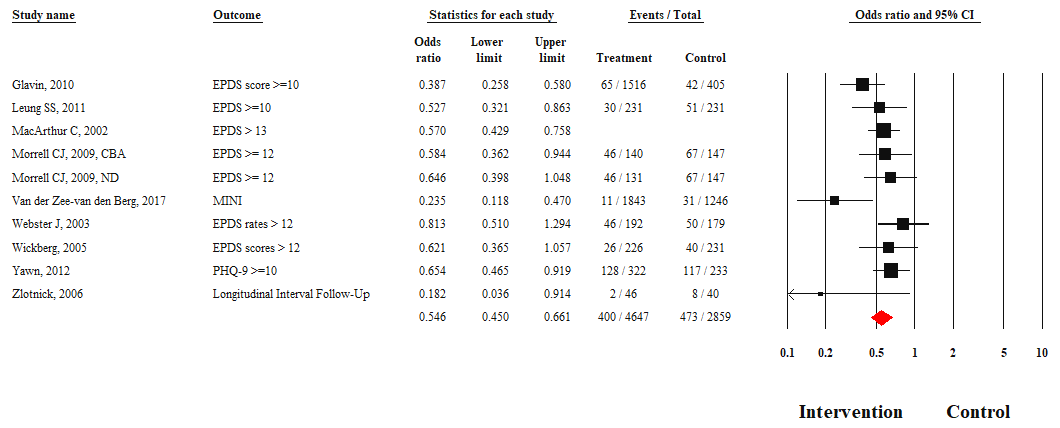


Supplementary figure 2: Effectiveness of screening programmes in perinatal state anxiety

Supplementary figure 3: Effectiveness of screening programmes in perinatal psychological quality of life

Supplementary figure 4: Effectiveness of screening programmes in perinatal physical quality of life

Supplementary figure 5: Effectiveness of screening programmes in improving treatment seeking behaviours

Supplementary figure 6: Funnel plot visualizing publication bias in reporting of depression outcome

Supplementary figure 7: Summary of risk of bias across studies


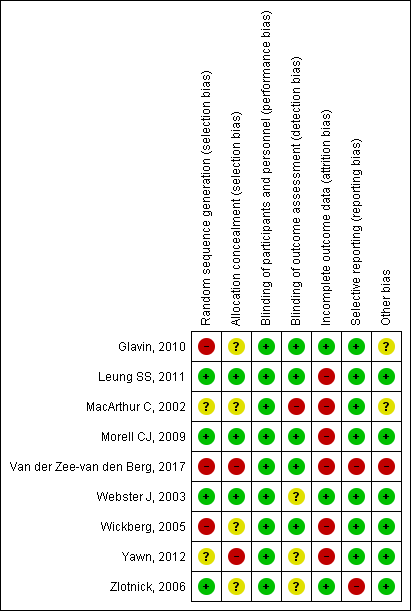

Supplement: Supplementary file 1 — Additional file 1: Supplementary Figure 1. Effectiveness of screening programmes in perinatal depression. Supplementary Figure 2. Effectiveness of screening programmes in perinatal state anxiety. Supplementary Figure 3. Effectiveness of screening programmes in perinatal psychological quality of life. Supplementary Figure 4. Effectiveness of screening programmes in perinatal physical quality of life. Supplementary Figure 5. Effectiveness of screening programmes in improving treatment seeking behaviours. Supplementary Figure 6. Funnel plot visualizing publication bias in reporting of depression outcome. Supplementary Figure7. Summary of risk of bias across studies. [file 12888_2022_3694_MOESM1_ESM.docx]
